# Anti-angiogenic effect of *Bryopsis plumosa*-derived peptide via aquaporin 3 in non-small cell lung cancer

**DOI:** 10.3892/ijo.2024.5711

**Published:** 2024-11-27

**Authors:** Heabin Kim, Seung-Hyun Jung, Seonmi Jo, Jong Won Han, Moongeun Yoon, Jei Ha Lee

**Affiliations:** 1 Department of Bio-material Research, National Marine Biodiversity Institute of Korea, Seocheon 33662, Republic of Korea; 2 Department of Biological Application & Technology, National Marine Biodiversity Institute of Korea, Seocheon 33662, Republic of Korea; 3 Department of Ecology & Conservation, National Marine Biodiversity Institute of Korea, Seocheon 33662, Republic of Korea

**Keywords:** anticancer peptide, angiogenesis, non-small cell lung cancer, zebrafish

## Abstract

Developing novel anti-angiogenic agents with minimal toxicity is notably challenging for cancer therapeutics. The discovery and development of peptides, whether derived from natural sources or synthesized, has potential for developing anti-angiogenic agents characterized by their ability to penetrate cancer cells, high specificity and low toxicity. The present study identified a *Bryopsis plumose*-derived anticancer and anti-angiogenesis marine-derived peptide 06 (MP06). A 22-amino acid peptide was synthesized and conjugated with fluorescein isothiocyanate (FITC-MP06) for intracellular localization in H1299 non-small cell lung cancer cells. Regulatory effects of this peptide on the viability, migration and self-renewal of lung cancer cells was assessed. Furthermore, anti-angiogenic effect of MP06 was investigated by monitoring vascular tube formation in human umbilical vein endothelial cells and a zebrafish model. Aquaporin (AQP)3, a membrane channel in various tissues, is involved in regulating stemness, epithelial-mesenchymal transition (EMT) and angiogenesis. MP06 downregulated AQP3 expression. Consistently, AQP3 knockdown by RNA silencing downregulated its gene expression, leading to a decrease in stemness, EMT and angiogenesis properties in H1299 cells. MP06 could thus serve as a novel therapeutic target with anticancer and angiogenesis properties for non-small cell lung cancer.

## Introduction

Lung cancer is the most prevalent malignant tumor among all types of cancer and a common cause of cancer-associated mortality (18.7% of all cancers globally). The incidence of lung cancer is considerably higher in males (32.1%) than in females (16.2% of age standardized rate globally), with smoking being a key contributing factor (1). Lung cancer is categorized into small and non-small cell lung cancer (NSCLC). NSCLC accounts for up to 85% of diagnosed lung cancer cases (2,3). Despite potent treatments, advanced-stage NSCLC poses a notable risk of recurrence, with 30-50% of patients experiencing disease progression following initial treatment (4). NSCLC cells aggressively proliferate, invade surrounding tissue, and cross the basement membrane, migrating to other organs in the body through the vascular or lymphatic systems (5) and leading to poor treatment outcomes, with 5-year survival rates for metastatic NSCLC remaining below 5% (6). Advances in molecular biology tools and biological processes have provided a comprehensive understanding of the fundamental biology of tumors (7). Lung cancer progression is associated with angiogenesis, and extensive angiogenesis is associated with invasion and poor prognosis. Hence, anti-angiogenic drugs present potential clinical efficacy in treating patients with lung cancer, prompting research into associated anti-angiogenic strategies (8,9).

Cells exhibiting cancer stem cell (CSC) properties serve a role in tumor initiation, perpetuation and advancement (10). CSCs are distinguished based on their differentiation and self-renewal capability (11) and considerably contribute to resistance against chemotherapy and radiotherapy (12,13). During cancer progression, CSCs can lead to tumor recurrence, which involves epithelial-mesenchymal transition (EMT). EMT represents phenotypical changes in cells transitioning from the epithelial to mesenchymal type, with high N-cadherin and vimentin expression in breast, lung, colon and head and neck carcinoma. EMT involves cell phenotype plasticity, contributing to intratumor heterogeneity (14,15). The potential association between EMT and CSCs is the key to drug resistance and cancer cell plasticity, which contributes to the development of cancer cells into malignant tumors (16,17).

Marine-derived peptide 06 (MP06), a 22-amino acid peptide derived from the green sea alga *Bryopsis plumosa*, leads to decreased proliferation in NSCLC while minimally affecting normal lung fibroblasts: Our previous study reported low metastatic potential of MP06 against lung cancer cells and zebrafish models (18). MP06 decreased the phosphorylation of ERK in A549 and H460 cells followed by downregulation of the ERK pathway (18). The small size (~50 amino acids) and high solubility of therapeutic potential peptides offer optimized pharmacokinetics, enhanced uptake in target tissue and more rapid removal from non-target tissues compared with other existing therapeutic agents such as antibodies and small molecule, making them well-suited for anticancer therapy (19). Numerous sources exhibiting endogenous antiangiogenic properties that regulate tumor growth and angiogenesis have been reported (20,21). The anti-angiogenic activities emphasize the potential of MP06 as an effective cancer therapeutic, in addition to its previously known anti-cancer properties (18).

Aquaporins (AQPs), a family of transmembrane water channel proteins, are widely distributed in various types of tissue and control water movements in extra- and intracellular fluid passages (22). AQPs serve key roles in physiological functions, including urine concentration, lactation and formation of tears, sweat and saliva (23-25). Additionally, increased and ectopic expression of certain types of AQP is associated with pathological manifestation and poor prognosis in several types of cancer (25). AQP3 is widely expressed in the normal respiratory tract and maintains water homeostasis. AQP3 inhibition increases sensitivity of prostate cancer cells to cryotherapy (26). Notably, AQP3 levels are associated with lung cancer progression, specifically maintenance of water homeostasis and differentiation of lung carcinoma (27). AQP3 expression is positively associated with angiogenesis in patients with NSCLC (28). Furthermore, suppressing AQP3 expression can inhibit cell proliferation and angiogenesis in human NSCLC xenografts (29). Recently, compared with non-neoplastic lung tissue, a notably high expression of AQP3 was found in lung adenocarcinoma samples (30). AQP3 expression affects lung cancer cell properties including proliferation, migration, metastasis and angiogenic potential (27-29). Therefore, the present study investigated the anticancer and anti-angiogenesis potential of MP06 in association with AQP3 expression in lung cancer cells and a zebrafish model. The findings may provide a novel potential therapeutic target for treating lung cancer metastasis and angiogenesis.

## Materials and methods

### Peptide synthesis

MP06 (LAV ISW KCQ EWN SLW KKR KRK T-NH2) and FITC-MP06 (with FITC tagged at the N-terminal) peptides (>95% purity) were synthesized by DANDI Cure Co. (Republic of Korea) through a solid-phase synthesis method. The molecular masses and purity of the peptides were analyzed using high-performance liquid chromatography as previously described (18). The synthesized peptides were prepared as a 10 mM stock solution in distilled water. For treatment, an aliquot of peptide stock solution was diluted RPMI-1640 medium (Invitrogen; Thermo Fisher Scientific, Inc.) supplemented with 10% fetal bovine serum and 1% antibiotics (penicillin/streptomycin; both HyClone; Cytiva). The 3D structure of MP06 was predicted using PEP-FOLD3 (bioserv.rpbs.univ-paris-diderot.fr/services/PEP-FOLD3) and PyMOL 3.0 software (pymol.org).

### Cell culture

The human H1299 lung cancer cell and MRC5 lung normal fibroblast cell lines (Korean Cell Line Bank; cat. no. 25803 and 10171) were cultured in RPMI-1640 medium supplemented with 10% fetal bovine serum and 1% antibiotics. Human umbilical vein endothelial cells (HUVECs; American Type Cell Culture, CRL-1730) were incubated in endothelial basal medium-2 (EBM; cat. no. CC-3156) containing endothelial cell growth medium supplements (cat. no. CC-4176; both Lonza). Cells were cultured at 37°C in a humidified 5% CO_2_ incubator. HUVECs were used between passages 3 and 4.

### Cytotoxicity assay and transfection

To investigate cytotoxicity, H1299 and human umbilical vein endothelial cells (HUVEC) were seeded at 5×10^3^ cells/well into a 96-well plate and treated with MP06 peptide at 5, 10 and 20 *μ*M at 37°C for 24 h. Subsequently, the medium was replaced with 100 *μ*l fresh RPMI-1640 and EBM containing 10 *μ*l Cell Counting Kit-8 (Dojindo Molecular). After 3 h, the absorbance was measured using the Spectramax i3x (Molecular Devices) at 450 nm. To suppress AQP3 expression, H1299 cells were transfected with 10 pmol small interfering (si)RNA)-AQP3 (cat. no. sc-29713; Santa Cruz Biotechnology) and negative siRNA control (cat. no. sc-37007) by Lipofectamine RNAi MAX (Invitrogen; Thermo Fisher Scientific, Inc.) at 37°C for 48 h according to the manufacturer's instructions. H1299 cells were cultured at 37°C in a humidified 5% CO_2_ incubator for at least 48 h after transfection.

### Reverse transcription (RT) PCR

To evaluate the expression of genes, total RNA from H1299 cells and HUVECs was isolated by TRIzol reagent (Invitrogen; Thermo Fisher Scientific, Inc.). The purity and quality of RNA were determined using a UV spectrophotometer. First-strand cDNA was synthesized using the cDNA synthesis kit (iNtRON Biotechnology). PCR was performed using AccuPower PCR preMix, Bioneer, S. Korea). Amplification with specific primers (Table I) was conducted as follows: Initial denaturation at 94°C for 5 min, followed by 30 cycles of denaturation at 94°C for 5 min, annealing at 56°C for 1 min and extension at 72°C for 1 min and final extension at 72°C for 3 min. The amplified PCR gene products were analyzed by 1% agarose gel electrophoresis containing redsafe (iNtRON Biotechnology) and imaged under UV light. β-actin was used as a reference gene.

### Western blotting

H1299 and HUVEC cells were lysed by vortexing with RIPA lysis buffer containing protease and phosphatase inhibitor cocktails (Sigma-Aldrich; Merck KGaA). The supernatant was centrifuged at 13,000 × g at 4°C for 30 min and protein concentration was measured using the Bradford assay (Bio-Rad Laboratories, Inc.). Equivalent amounts (30 *μ*g) of protein were boiled for 5 min and separated on 10% SDS-PAGE and transferred to PVDF membranes. After blocking the membranes with a solution of tris-buffered saline (TBS) containing bovine serum albumin (BSA, 1%, Sigma-Aldrich; Merck KGaA; cat. no. A3294) at room temperature for 1 h and incubated overnight at 4°C with primary antibodies (all 1:1,000) against SOX2 (cat. no. sc-365823), Octamer binding transcription factor (OCT)3/4 (cat. no. sc-5279), Kruppel-like factor (KLF)4 (cat. no. sc-365144), CD44 (cat. no. sc-7297), N-cadherin (cat. no. sc-59987), E-cadherin (cat. no. sc-8426), Zinc-finger E-box-binding homeobox (ZEB)1 (cat. no. sc-515797), vimentin (cat. no. sc-6260), Snai1 (cat. no. sc-271977), AQP3 (cat. no. sc-518001) and β-actin (cat. no. sc-47778; all Santa Cruz Biotechnology) and VEGF (Bioswamp; cat. no. PAB30976). After 1 h incubation at room temperature with HRP-conjugated secondary antibodies (1:10,000; cat. nos. sc-2748 and rabbit sc-2357, Santa Cruz Biotechnology), membranes were rinsed with Tris-buffered saline and visualized using a western blotting substrate (Thermo Fisher Scientific, Inc.; cat. no. A38555).

### Immunofluorescence

H1299 cells (5×10^4^) were seeded on cover glass in cell culture plates. The cells were fixed using 4% paraformaldehyde for 30 min at room temperature, washed and blocked with phosphate-buffered saline (PBS) containing 1% BSA (Sigma-Aldrich; Merck KGaA) at room temperature for 40 min. Cells incubated with anti-SOX2 (cat. nosc-365823; 1:500) and anti-vimentin (cat. no. sc-6260; 1:500) in a solution of PBS at 4°C overnight. Then, the cover glass was washed with PBS and incubated with Alexa Fluor 488-conjugated antibody (Invitrogen; Thermo Fisher Scientific, Inc.; cat. no. A21202; 1:1,000) for 1 h at room temperature. Then cells were mounted with aqueous mounting containing DAPI at room temperature for 5 min (Vectashield Mounting Medium with DAPI H-1,200; Vector Laboratories). Cell images were acquired using a Zeiss LSM510 Meta fluorescence microscope at 40X magnification with ZEN 3.1 software. (Carl Zeiss GmbH).

### Tumor sphere forming assay

Stem cell-permissive medium was prepared with DMEM-F12 (Cat. No. 11320-033; Invitrogen) supplemented with 20 ng/ml epidermal growth factor (E9644; Sigma-Aldrich), 20 ng/ml basic fibroblast growth factor (13256-029; Invitrogen) and B27 serum-free supplement (Gibco; Thermo Fisher Scientific, Inc). AggreWell 400(STEMCELL, #34415) or 800(STEMCELL, #34425) microwell plates were pretreated and washed with anti-adhesion solution(STEMCELL, #07010) for 5 min at 37°C. Then, H1299 cells were seeded at 1×10^5^ cells/well and centrifuged at 100 × g for 3 min at room temperature to capture cells inside the microwells with stem cell-permissive medium. Cells were incubated at 37°C with 5% CO_2_ for 7-10 days. The formed H1299 spheroids were imaged using an inverted phase contrast microscope (Olympus Corporation; CKX53 light microscope; magnification, ×100).

### Wound healing assay

For wound healing assay, H1299 (1×10^5^) cells were seeded on a 6-well plate. When cells reached 80% confluence, a wound was introduced across the diameter of each well using a 200-*μ*l pipette tip. Images were captured by inverted phase contrast light microscopy, after 12 and 24 h in serum-free RPMI-1640 media with MP06 peptide. The healing area was quantified using ImageJ 1.54g software (National Institutes of Health).

### Invasion and migration assay

The migration and invasion assay was conducted using a Transwell chamber (8-*μ*m pores; BD Biosciences) in a 24-well plate. H1299 (2×10^4^) cells were seeded in the upper chambers with 200 *μ*l serum-free RPMI-1640 medium with or without MP06 peptide at 37°C in a humidified 5% CO_2_ incubator for 24 h. The lower chamber contained 500 *μ*l RPMI-1640 medium containing 10% FBS and 1% penicillin/streptomycin. For the invasion assay, a Transwell chamber was coated with diluted Matrigel (Corning, Inc.) for 30 min at 37°C. The migratory and invasive cells from the upper chamber were fixed with 4% paraformaldehyde for 20 min at 37°C and stained with crystal violet for 5 min at room temperature. The upper surface of Transwell membrane was wiped using a cotton swab to remove non-migratory and -invasive cells. Cells were then imaged using an inverted light microscope (Olympus CKX53; magnification, ×40).

### Tube formation assay

HUVECs (1×10^4^) were seeded to 80% confluency for final passage at passage 2 into a 96-well plate. Each well was coated with 50 *μ*l Geltrex matrix (Gibco; Thermo Fisher Scientific, Inc.) and allowed to solidify at 37°C for 30 min. H1299 culture media was collected, supernatant was centrifuged at 13,000 × g for 10 min at 4°C. Conditioned media (CM) were produced using mixed fresh EBM media/H1299 cultured media with MP06 or siAQP3 ratios (75:25, 50:50). The HUVECs were incubated at 37°C with 5% CO_2_ for 24 h and stained with Calcein-AM (Invitrogen) at 37°C for 5 min. Angiogenesis was observed using an inverted light microscope at 100X magnification.

### Zebrafish vascular tube formation

Zebrafish (*Danio rerio*) were provided by Professor C-H. Kim (Chungnam National University) and maintained as described in a previous study (31). Wild-type and transgenic (*kdrl*:eGFP) embryos were obtained by breeding males and females (2:2) in a 14/10-h light/dark cycle at 28.5°C with a recirculating water system. For anti-angiogenesis assay, fertilized zebrafish embryos were transferred to a 24-well plate (10 specimens/well) at the 70% epiboly stage. Embryos were exposed to 2 *μ*M MP06 peptide by dissolving in egg water (60 *μ*g/ml sea salt in distilled water). The zebrafish embryos were incubated for 24 h at 28.5°C. Embryos were anesthetized and mounted in 3% methylcellulose (Sigma-Aldrich; Merck KGaA) and then photographed using a fluorescence microscope at 40X magnification. (Leica DM6 B; Leica GmbH).

### Gene expression profiling and Kaplan-Meier plotter

The AQP3 of expression profile of lung adenocarcinoma in tumor and normal tissue was analyzed from Gene Expression Profiling Interactive Analysis web server (gepia.cancer-pku.cn/detail. php?gene=AQP3, accessed April 24, 2024). This web server extracts data from TCGA data portal and Genotype-Tissue Expression (GTEx) database of normal tissue. Kaplan-Meier survival was analyzed using Gene Expression database of lung normal and tumor tissues 2 on expression of AQP3 (gent2.appex.kr/gent2/, accessed March 24, 2024).

### Statistical analysis

Data are presented as the mean ± standard error of the mean. All experiments were conducted in triplicate. Comparisons were performed using two-tailed unpaired Student's t test or one-way ANOVA and Tukey's post hoc test for multiple comparisons. GraphPad Prism 10.3.1 (Dotmatics) was used for statistical analysis. P<0.05 was considered to indicate a statistically significant difference.

## Results

### Structure of MP06 and cell viability

**Figure 1:**
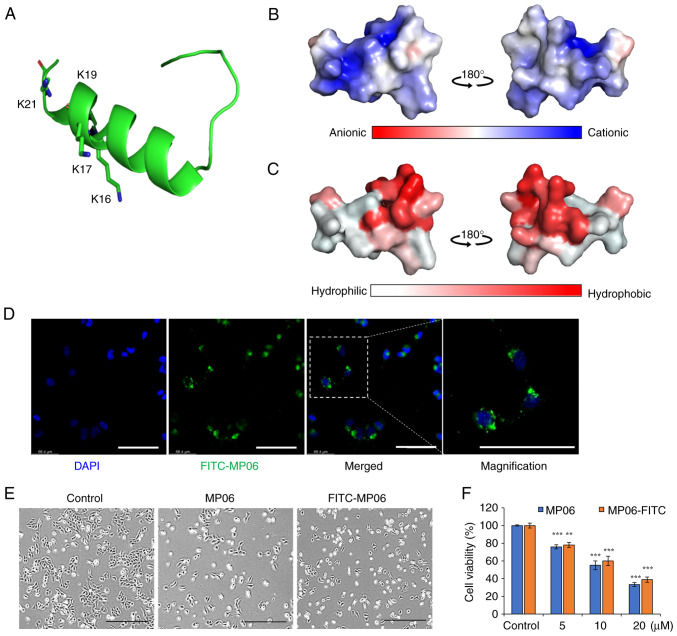
3D structure of MP06 and cell viability. (A) MP06 contains an N-terminal α-helix and unstructured C-terminal region. Basic amino acids contribute to positive net charge of MP06. (B) Electrostatic potential of MP06. Blue and red regions are the positively and negatively charged regions. (C) Hydrophobic and hydrophilic regions of MP06 are visualized as red and white. (D) FITC-MP06 accumulation was observed in the cytoplasm of H1299 cells. Scale bar, 100 *μ*m. (E) Morphological changes from a spindle shape to a cobblestone-like shape in H1299 cells induced by MP06 and FITC-MP06 peptides. Scale bar, 500 *μ*m. (F) Cell viability. ^**^P<0.01, ^***^P<0.001; vs. control group. MP06, marine-derived peptide 06; con, control.

MP06 tertiary structure was predicted using PEP-FOLD3 modeling (32). MP06 contained an N-terminal α-helix, unstructured C-terminal region and several basic amino acids that make its net charge positive (Fig. 1A and B). The charged amino acids were concentrated on one side of the helix, whereas hydrophobic amino acids formed the other side (Fig. 1C). FITC-MP06 was synthesized to examine its cellular effects and distribution (Fig. S1). FITC-MP06 accumulation was observed in the cytoplasm of H1299 cells (Fig. 1D). Morphological changes from spindle shape to a cobblestone-like shape were observed in cells treated with 20 *μ*M MP06 or FITC-MP06 when compared to the untreated control cells (Fig. 1E). There was a dose-dependent decrease in viability in cells treated with MP06 or FITC-MP06, with a decrease in the cell viability of up to ~38% of the cells treated with MP06 (Fig. 1F). Collectively, these results indicated that MP06 and FITC-MP06 efficiently infiltrated cellular membranes, were localized in cytoplasm, and increased cytotoxicity in lung cancer cells.

### MP06 regulates stemness of lung cancer cells

**Figure 2:**
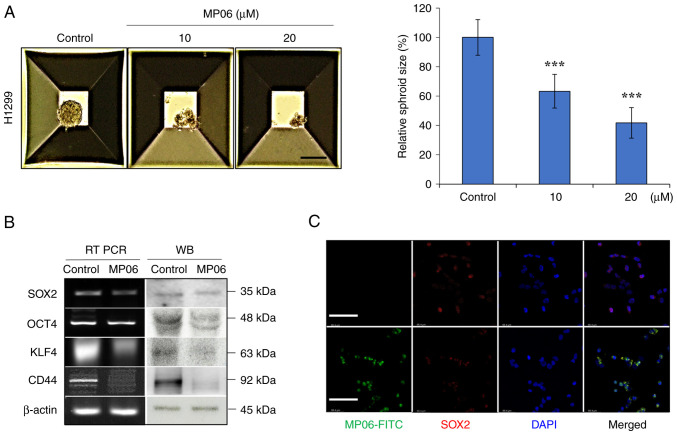
Regulation of stemness potential and sphere forming ability induced by MP06 in lung cancer cells. (A) Sphere forming assay of H1299 cells treated with MP06. (B) Cell levels of CSC markers in H1299 cells with MP06 using RT PCR and WB. (C) Immunofluorescence staining for expression of SOX2 and FITC-MP06. Scale bar, 100 *μ*m. ^***^P<0.001 vs. control. MP06, marine-derived peptide 06; RT, reverse transcription; WB, western blotting; OCT, octamer binding transcription factor; KLF, Kruppel-like factor.

CSC transcription factors (such as SOX2, OCT4, KLF4 and CD44) are important marker of cancer and normal stem cells (35,36). Following treatment with MP06, spheroid size significantly decreased compared with control H1299 cells (Fig. 2A). MP06 decreased expression of stemness markers (SOX2, OCT4, KLF4 and CD44), which indicated decreased self-renewal potential (Fig. 2B) Suppressed SOX2 expression was detected by immunofluorescence in FITC-MP06-treated H1299 cells (Fig. 2C). The results showed that MP06 partly regulated suppression of self-renewal activity in H1299 cells.

### MP06 is involved in reducing EMT

**Figure 3:**
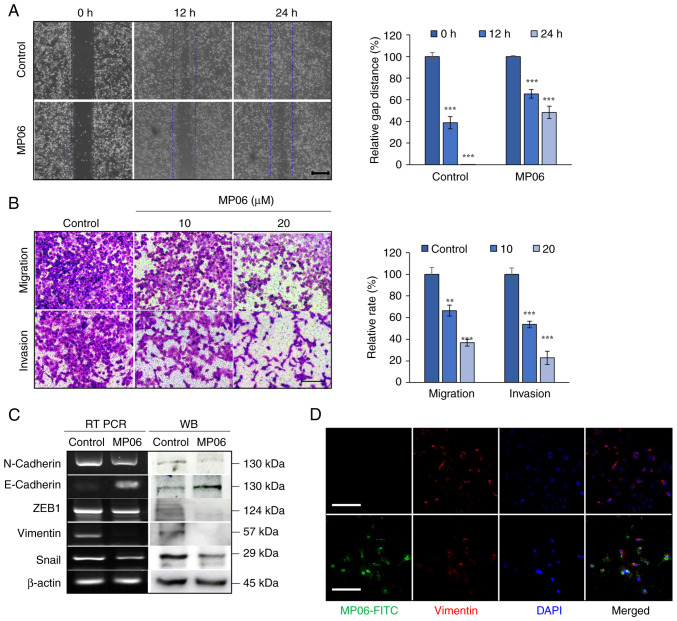
Regulation of EMT and migratory and invasive activity of MP06 in H1299 cells. (A) Wound healing in H1299 cells treated with MP06. (B) Migration and invasion abilities of H1299 cells following treatment with MP06. Scale bar, 500 *μ*m. (C) Cellular levels of EMT markers in H1299 cells treated with MP06. (D) Expression of vimentin and FITC-MP06 through immunofluorescence staining. Scale bar, 100 *μ*m. ^**^P<0.01, ^***^P<0.001 vs. control. EMT, Epithelial-mesenchymal transition; MP06, marine-derived peptide 06; RT PCR, reverse transcription-PCR; WB, western blotting; ZEB, Zinc-finger E-box-binding homeobox.

To investigate the metastatic function of MP06 on H1299 cells, EMT-associated properties were investigated by wound healing, migration and invasion assay. A significant decrease in the wound gap closure was observed after MP06 treatment (Fig. 3A). MP06-treated cells exhibited a significant decrease in migration and invasion abilities (Fig. 3B). MP06 notably decreased the cellular levels of important EMT markers (such as N-cadherin, ZEB1, vimentin and Snail) that regulate the migration of mesenchymal cells. E-cadherin, a representative marker of epithelial cells, expression increased (Fig. 3C). Reduced expression of vimentin was confirmed by immunofluorescence staining in FITC-MP06-treated H1299 cells (Fig. 3D). Collectively, these results suggested the involvement of MP06 in migration and invasion via decreased EMT marker expression in H1299 cells.

### MP06 inhibits angiogenesis by downregulating VEGF

**Figure 4:**
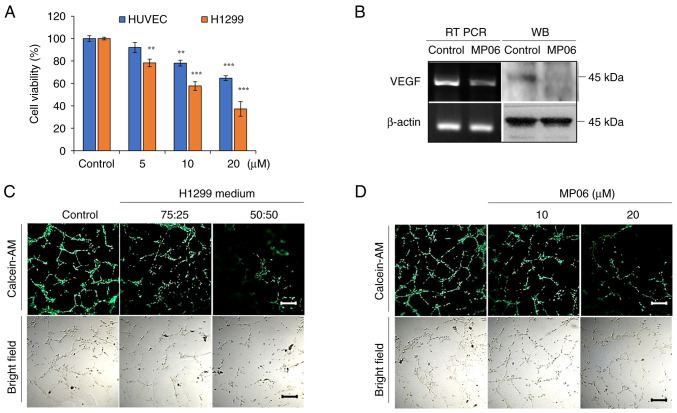
Suppression of angiogenesis and expression of VEGF induced by MP06. (A) Cytotoxicity of H1299 cells and HUVECs treated with MP06. (B) Expression of VEGF in H1299 cells treated with MP06. (C) Tube formation ability of HUVECs in conditioned medium (fresh EBM media/H1299 cultured media with MP06 ratios(75:25, 50:50). (D) Formation of tube-like structures in HUVECs reduced by MP06. Scale bar, 500 *μ*m. ^**^P<0.01, ^***^P<0.001 vs. control. VEGF, vascular endothelial growth factor; HUVEC, Human Umbilical Vein Endothelial Cells; RT, reverse transcription; WB, western blotting; MP06, Marine peptide 06.

Effective angiogenesis is key for the growth of the primary tumor and the cancer cells' ability to spread to other locations in the body. To investigate the angiogenic ability of MP06, HUVECs, which can form tube-like structures depending on the VEGF content in the supernatant (38), were used. MP06-induced cytotoxicity in HUVECs was significantly lower than that in H1299 cells (Fig. 4A). MP06 notably decreased VEGF expression in H1299 cells (Fig. 4B). MP06-CM treatment decreased tube formation compared with that in the control H1299 culture media, implying that MP06 suppressed angiogenesis activity at a high rate with MP06-CM (Fig. 4C). The formation of tube-like structures and expression of VEGF in HUVECs was directly inhibited by MP06-treated EBM (Figs. 4D and S2). These results suggested the involvement of MP06 in VEGF-mediated tube formation and blockade of angiogenic responses.

### MP06-mediated suppression of angiogenesis in zebrafish embryos

**Figure 5:**
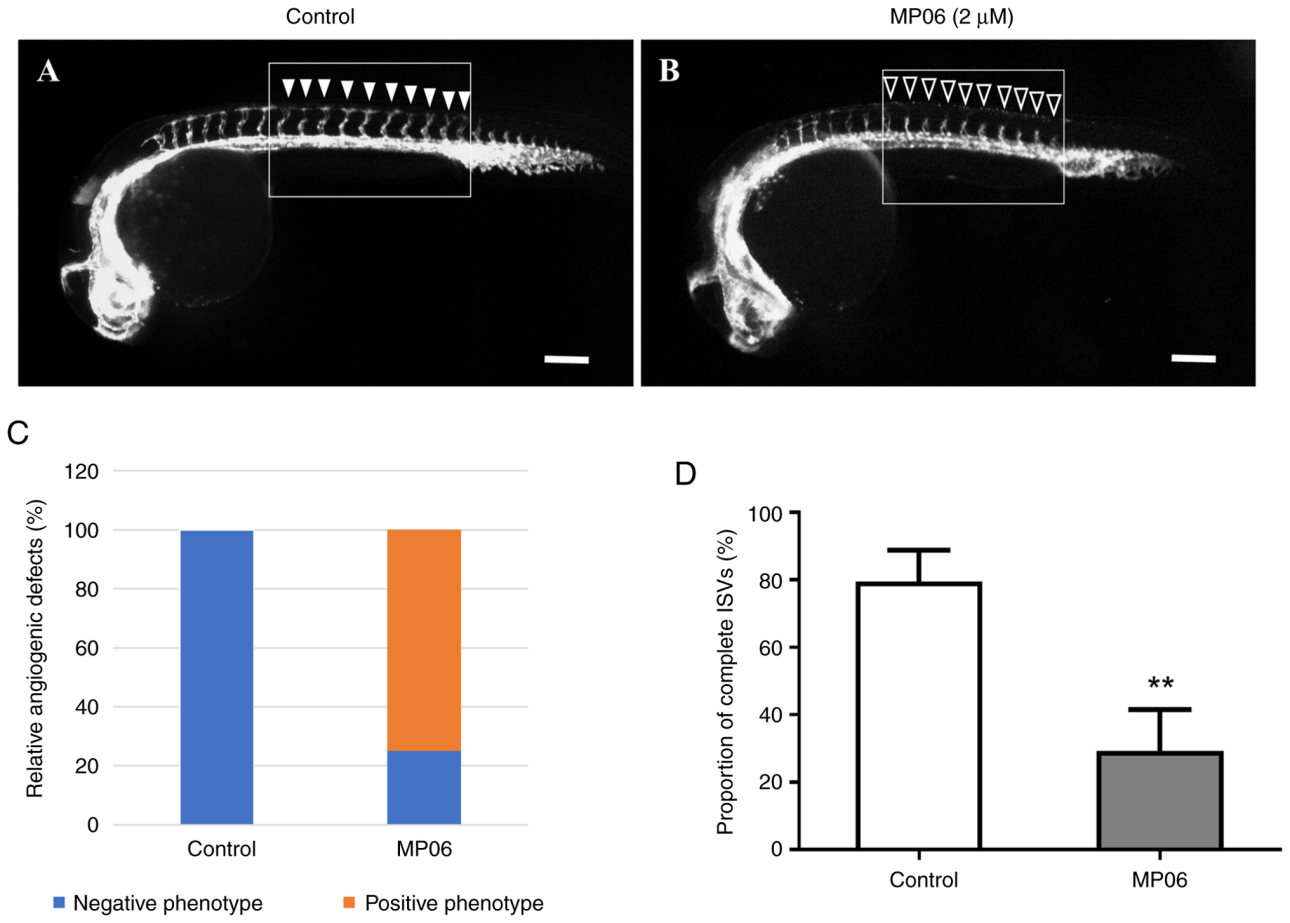
MP06-mediated suppression of angiogenesis by MP06 in zebrafish embryos. Lateral view of (A) untreated and (B) MP06-treated embryos exhibit reduced growth of ISVs. (C) Quantitative comparative analysis of the angiogenic effects in the control group and the treated with MP06 group. positive phenotype showing enhanced ISV growth (solid arrows) and the negative phenotype (hollow arrows). (D) Proportion of completed ISV structure in MP06-treated zebrafish at 30 h post-fertilization compared with untreated controls scale bar, 200 *μ*m. ^**^P<0.01 vs. control. MP06, Marine peptide 06; ISV, intersegment vessels.

MP06 can be crucial for angiogenesis potential of zebrafish embryos. A concentration of 2 *μ*M MP06 was used to observe inhibition of angiogenesis, as concentrations >4 *μ*M resulted in zebrafish embryo death. Changes in vascular patterning following MP06 treatment were observed during zebrafish development, particularly in transgenic zebrafish expressing the Tg (kdrl:eGFP) marker. MP06-treated embryos exhibited reduced growth of intersegment vessels (ISVs) at the apex compared with untreated control embryos in lateral view (Fig. 5A and B). In untreated control embryos, robust ISV growth was observed (filled arrowheads) compared with MP06-treated embryos, which showed noticeably decreased ISV growth (empty arrowheads) at the top of the embryo (Fig. S3). Quantification of the proportion of completed ISV structures in MP06-treated zebrafish at 30 h post-fertilization revealed a significant decreased of ~2.7-fold compared with that in untreated controls, indicating an adverse effect of MP06 treatment on vascular development (Fig. 5C and D). These angiogenic events, accompanied by decreased proliferation and migration in endothelial cell, support the role of MP06 as a negative regulator of angiogenesis.

### Expression of AQP3 in H1299 cells

**Figure 6:**
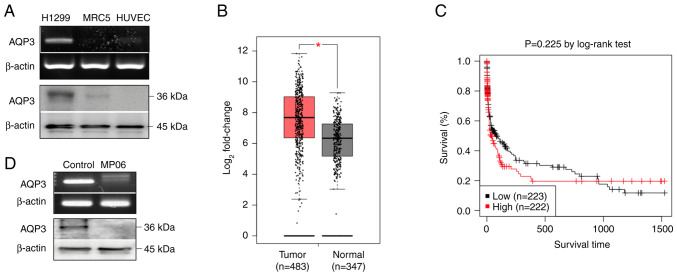
Expression of AQP3 in lung cancer. (A) Levels of AQP3 in lung cancer (H1299) and normal cells (MRC5 and HUVEC). (B) Gene expression analysis of AQP3 in normal and cancer lung tissue using Gene Expression Profiling Interactive Analysis. ^*^P<0.05 (C) Kaplan-Meier survival graph of patients with lung cancer according to AQP3 expression. AQP, aquaporin; HUVEC, human umbilical vein endothelial cell.

Expression levels of AQP3 gene and protein were higher in H1299 cells than in normal cells (MRC5 cells and HUVECs; Fig. 6A). Furthermore, it was confirmed that AQP3 was more highly expressed in lung cancer than in normal lung tissue using Gene Expression Profiling Interactive Analysis web server (gepia.cancer-pku.cn/detail.php?gene=AQP3, accessed April 24, 2024; Fig. 6B). Gene expression profiling revealed that AQP3 was markedly upregulated in lung adenocarcinoma tissue. Additionally, the Kaplan-Meier survival analysis using Gene Expression database of normal and tumor tissues 2 (gent2.appex.kr/gent2/, accessed March 24, 2024) revealed an association between AQP3 and lung cancer. The Kaplan-Meier graph confirmed that the survival rate of patients with lung cancer and high AQP3 expression was low (Fig. 6C). H1299 cells treated with MP06 showed decreased levels of AQP3 expression (Fig. 6D). These results indicated that high AQP3 expression was associated with malignancy and decreased survival in lung cancer.

### Regulation of stemness, EMT and angiogenesis by downregulating AQP3

**Figure 7:**
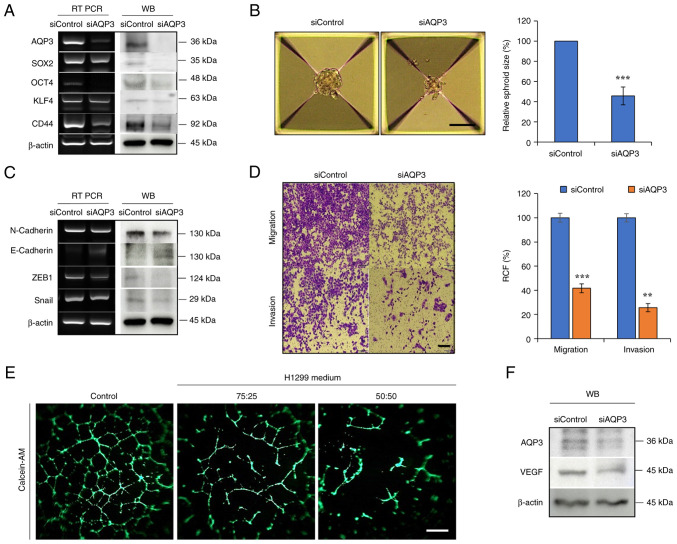
Regulation of stemness, EMT and angiogenesis by downregulating AQP3. (A) Expression of AQP3 and cancer stem cell markers in AQP3-knockdown H1299 cells. (B) Sphere-forming ability in AQP3-knockdown H1299 cells. Scale bar, 100 *μ*m. (C) Expression of AQP3 and EMT markers in AQP3-knockdown H1299 cells. (D) Migratory and invasive ability of AQP3-knockdown H1299 cells. Scale bar, 200 *μ*m. (E) Angiogenesis activity suppressed by conditioned medium (fresh EBM media/H1299 cultured media with siAQP3 ratios (75:25, 50:50). (F) VEGF expression. ^**^P<0.01, ^***^P<0.001. EMT, epithelial-mesenchymal transition; AQP, aquaporin; RT, reverse transcription; WB, western blotting; si, small interfering; OCT, octamer binding transcription factor; KLF, Kruppel-like factor; VEGF, vascular endothelial growth factor; RCF, relative colony forming; ZEB, Zinc-finger E-box-binding homeobox.

AQP3 gene was commonly overexpressed in H1299 cells compared with normal cells (Fig. 6A). AQP3 was knocked down to determine its effects on stemness, EMT and angiogenesis-associated changes in H1299 cells. Expression of the marker proteins SOX2, OCT4, KLF4 and CD44 was decreased following AQP3 knockdown, which was consistent with the decreased spheroid size (Fig. 7A and B). AQP3 knockdown decreased expression of EMT markers such as N-cadherin, ZEB1 and Snail, accompanied by decreased migration/invasion of H1299 cells (Fig. 7C and D). siAQP3-CM decreased tube formation compared with that in control H1299 culture medium, suggesting that AQP3 knockdown suppressed angiogenesis activity in siAQP3-CM-cultured cells (Fig. 7E). VEGF secretion regulates CSC and EMT phenomena through autocrine action (36). Consistent with VEGF expression in MP06-treated H1299 cells, VEGF expression consistently decreased in H1299 cells treated with siRNA-AQP3 (Fig. 7F).

Altogether, the *in vitro* and *in vivo* data suggested that MP06 was partially associated with cancer stemness and EMT in H1299 cells and that suppressing AQP3 expression to target VEGF may be an effective anti-angiogenic therapy.

## Discussion

Excessive angiogenesis notably contributes to cancer progression by supplying key nutrients and oxygen to support tumor growth and metastasis (2,5). Thus, development of novel antiangiogenic therapeutic agents that exhibit high effectiveness and few side effects is crucial. Effectiveness of conventional therapy is often limited by drug resistance and lack of specificity (3,4). Peptides have emerged as key therapeutic agents for the study of angiogenesis-dependent disease because of efficient penetration of cancer cells, high specificity and low toxicity. Many anti-angiogenic proteins are large and complex and have limited tissue penetration ability, making their production at therapeutic volumes costly (38,39). However, peptides have garnered interest as anti-angiogenic candidates owing to specific advantages, such as smaller size, easier tissue penetration and lower production costs compared with proteins and antibodies (18,19). Anticancer peptides (including Tebentafusp, Buserelin, Plitidepsin, Triptorelin, and Dactinomycin), serve key roles in cancer treatment, positioning them as promising future therapeutics (38,40). MP06 had a hydrophilic/phobic ratio of 59% and a net charge of +6. Because of this positive charge, it interacts electrostatically with the negatively charged membrane, enhancing attachment and activity during membrane permeation. The cationic amphipathic helical structure of MP06 was consistent with that of other anticancer peptides, such as GI-15 and A12L/A20L. Anticancer and antimicrobial peptides exert their activity through characteristic structural features (33,34). A. The potential role of MP06 was investigated based on its promising anticancer anti-angiogenic activities in NSCLCs. MP06 exhibits lower toxicity in normal lung fibroblasts and HUVECs than in H1299 cells and is soluble in water, making it a potential candidate for drug development (18). Hemolysis is not observed in horse erythrocytes treated with MP06 at ≥100 *μ*M, indicating MP06 efficiently penetrates horse erythrocytes without cytotoxic effects.

CSCs modulate extracellular matrix (ECM) and use intracellular signaling pathways to maintain homeostatic processes such as EMT and angiogenesis (41). ECM-mediated changes in the expression and/or cellular localization of SOX2, OCT4 and KLF4 are associated with prostate and breast cancer (35,36). The ECM microenvironment can revert non-tumorigenic cells into CSCs via EMT-associated processes, thereby increasing cell invasion and metastasis (42,43). Here, MP06 suppressed migration and invasion, which are key factors in reducing tumorigenicity in H1299 cells. However, whether MP06 regulates cellular migration and cancer stemness is unclear. Further studies are required to investigate whether MP06 influences the expression of genes associated with migration and CSCs.

Angiogenesis results from an imbalance between pro- and anti-angiogenic endogenous factors that contribute to disease progression (8). The key factors include VEGF, fibroblast and platelet-derived growth factor and angiopoietins, which interact with the ECM. The interaction between ECM and endothelial cells is crucial for various cellular processes in many cancers, including NSCLC, gastric, and uterine cancer (9). The role of VEGF in angiogenesis makes it a promising target for cancer therapy. However, the clinical use of VEGF-targeted therapies is hindered by the potential side effects such as hypertension, proteinuria, bleeding, and cardiovascular complications in achieving optimal therapeutic concentrations (44,45). The present study ascertained the effects of anti-angiogenesis of MP06 and its anticancer properties using a zebrafish embryo model for screening. MP06 decreased gene and protein levels of VEGF in H1299 and HUVECs, which was associated with vessel formation of zebrafish embryos. Suppression in vascular patterning following MP06 treatment were observed during zebrafish development. Strategies such as controlled release of VEGF from ECM scaffolds may improve the efficacy and safety of anti-angiogenic therapy (8,9).

AQPs regulate vascular formation and proliferation through VEGF within tumors and offer targets for cancer therapy (23-25). Cell migration and regulation of angiogenesis are suppressed following AQP5 knockdown via the EGFR/ERK signaling pathway (46). Similarly, downregulation of AQP3 inhibits proliferation via the hypoxia-inducible factor (HIF)-1a/VEGF and ERK pathways in NSCLC (47). AQP3, 4 and 5 are expressed in H1299 cell line, derived from the lymph nodes and has been widely used to investigate various disease-associated tumor metastases (29,46). The present study demonstrated that AQP3 was more highly expressed in lung cancer than in normal tissues. AQP3 serves pivotal roles in NSCLC progression, migration and angiogenesis (27,29,48). AQP3 is associated with maintenance of stemness not only in CSCs but also in normal stem cells (49,50). AQP3 promotes stem cell-like properties by regulating AQP3/STAT3/CD133 expression in hepatocellular carcinoma cells (51). AQP3 serves a key role in the progression and metastasis of various types of cancer: AQP3 can upregulate matrix metalloproteinases (MMP1, MMP2 and MMP9) and induce EMT by activating the PI3K/AKT signaling in gastric cancer (52,53). A previous study indicated that MP06 suppresses the ERK signaling pathway, which regulates cancer cell migration and proliferation in NSCLC (18). The regulation of these signaling pathways may facilitate cancer treatment by inhibiting tumor-specific angiogenesis. The present study suggested downregulation of AQP3 may suppress tumor-specific vascularization. However, the direct association between MP06 and AQP3 remains elusive and further studies are required to verify the role of VEGF *in vivo*.

The multifaceted approach of targeting angiogenesis and EMT signaling holds promise for development of effective cancer therapy with minimal toxicity. Further research on the mechanisms of action and clinical translation of therapeutic strategies are warranted to improve cancer treatment outcomes. Collectively, the present study showed that MP06 may decrease AQP3 expression and serve as a new target for suppressing angiogenesis in NSCLC.

## Supplementary Data



## Figures and Tables

**Table I:** Primer sequences for reverse transcription PCR.

Primer	Sequence, 5′→ 3′)
SOX2-F	CAAGATGCACAACTCGGAGA
SOX2-R	TTCATGTGCGCGTAACTGTC
OCT4-F	TGGGATATACACAGGCCGAT
OCT4-R	GTGACAGAGACAGGGGGAAA
KLF4-F	CCCACCTTCTTCACCCCTAGA
KLF4-R	CCCAGTCACAGTGGTAAGGTT
CD44-F	TCATACCAGCCATCCAATGC
CD44-R	CGTGTGTGGGTAATGAGAGG
β-actin-F	CTTCGCGGGCGACGAT
β-actin-R	CCACATAGGAATCCTTCTGA
N-cad-F	ACTTGCCAGAAAACTCCAGG
N-cad-R	TGGTGTATGGGGTTGATCCT
E-cad-F	TGGATAGAGAACGCATTGCC
E-cad-R	AAAATCCAAGCCCGTGGTG
ZEB1-F	CGGCGCAATAACGTTACAAA
ZEB1-R	AAAGGTGTAACTGCACAGGG
Vimentin-F	GAGAACTTTGCCGTTGAAGC
Vimentin-R	TCTGCTGGTATATGAGTGCTG
Snail-F	GGGACTGTGAGTAATGGCTG
Snail-R	CCCACTCCTCTATGACACCA
VEGF-F	ATCGAGACCCTGGTGGACA
VEGF-R	CCTCGGCTTGTCACATCTGC
AQP3-F	CCCTTATCGTGTGTGTGCTG
AQP3-R	TCAGCTGGTACACGAAGACA

OCT, octamer binding transcription factor; KLF, Kruppel-like factor; cad, cadherin; ZEB, Zinc-finger E-box-binding homeobox; VEGF, vascular endothelial growth factor; AQP, aquaporin; F, forward; R, reverse.

## Data Availability

The data generated in the present study may be requested from the corresponding author.

## Abbreviations

AQP: aquaporin
MP06: marine-derived peptide 06
HUVEC: human umbilical vein endothelial cell
EMT: epithelial-mesenchymal transition
NSCLC: non-small-cell lung cancer
CSC: cancer stem cell
Oct: octamer binding transcription factor
KLF4: Kruppel-like factor 4
WB: western blotting
Zeb1: zinc-finger E-box-binding homeobox 1
VEGF: vascular endothelial growth factor
siRNA: small interfering RNA
ISV: intersegment vessel
CM: conditioned medium
ECM: extracellular matrix
