# Supplementary material for: Anti-angiogenic effect of Bryopsis plumosa-derived peptide via aquaporin 3 in non-small cell lung cancer
Source: Int J Oncol. 2024 Nov 27;66(1):5. doi: 10.3892/ijo.2024.5711 (PMC11637497; doi:10.3892/ijo.2024.5711)

Figure S1. HPLC profiles and data of molecular masses of purified peptides. (A) Chromatographic profiles and (B) mass spectra for MP06 peptide and FITC-MP06. MP06, Marine peptide 06.

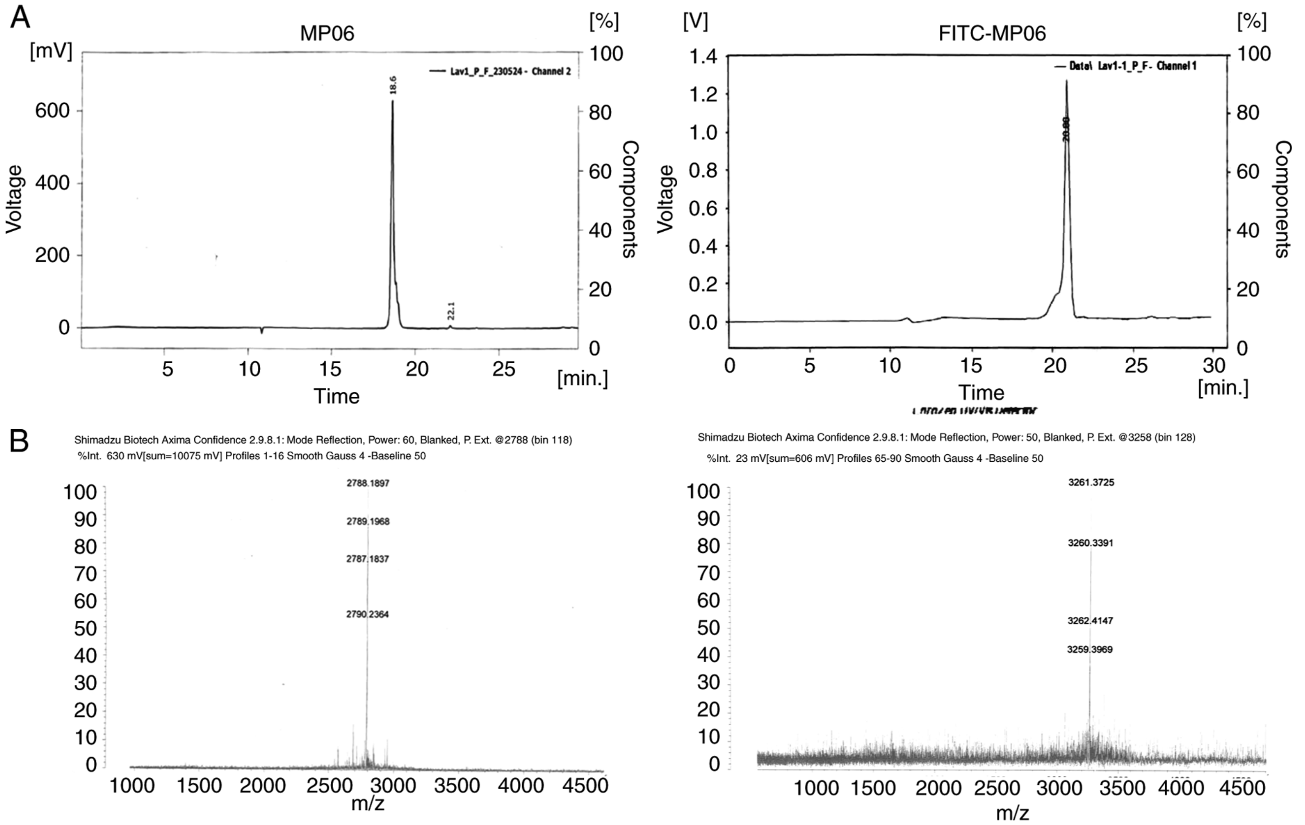

Figure S2. Expression of VEGF in Human Umbilical Vein Endothelial Cells treated with MP06 peptide. VEGF, vascular endothelial growth factor; MP06, Marine peptide 06.

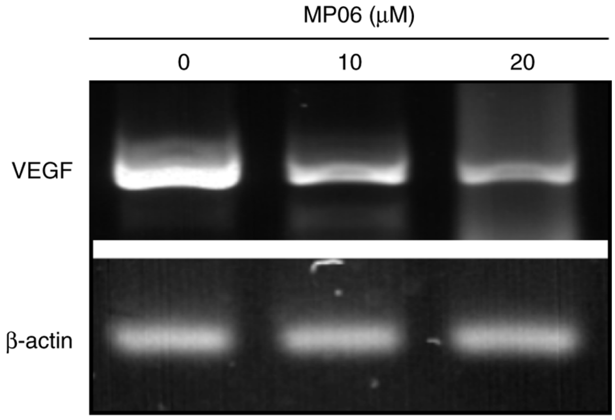

Figure S3. Vascular tube forming potential of MP06 peptide on zebrafish embryos in lateral view. In untreated control groups, robust ISV growth was observed (filled arrowheads) compared with 2 μm MP06-treated embryos, which showed decreased ISV growth (empty arrowheads) at embryos (scale bar, 200 μm). ISV, intersegment vessels; MP06, Marine peptide 06.

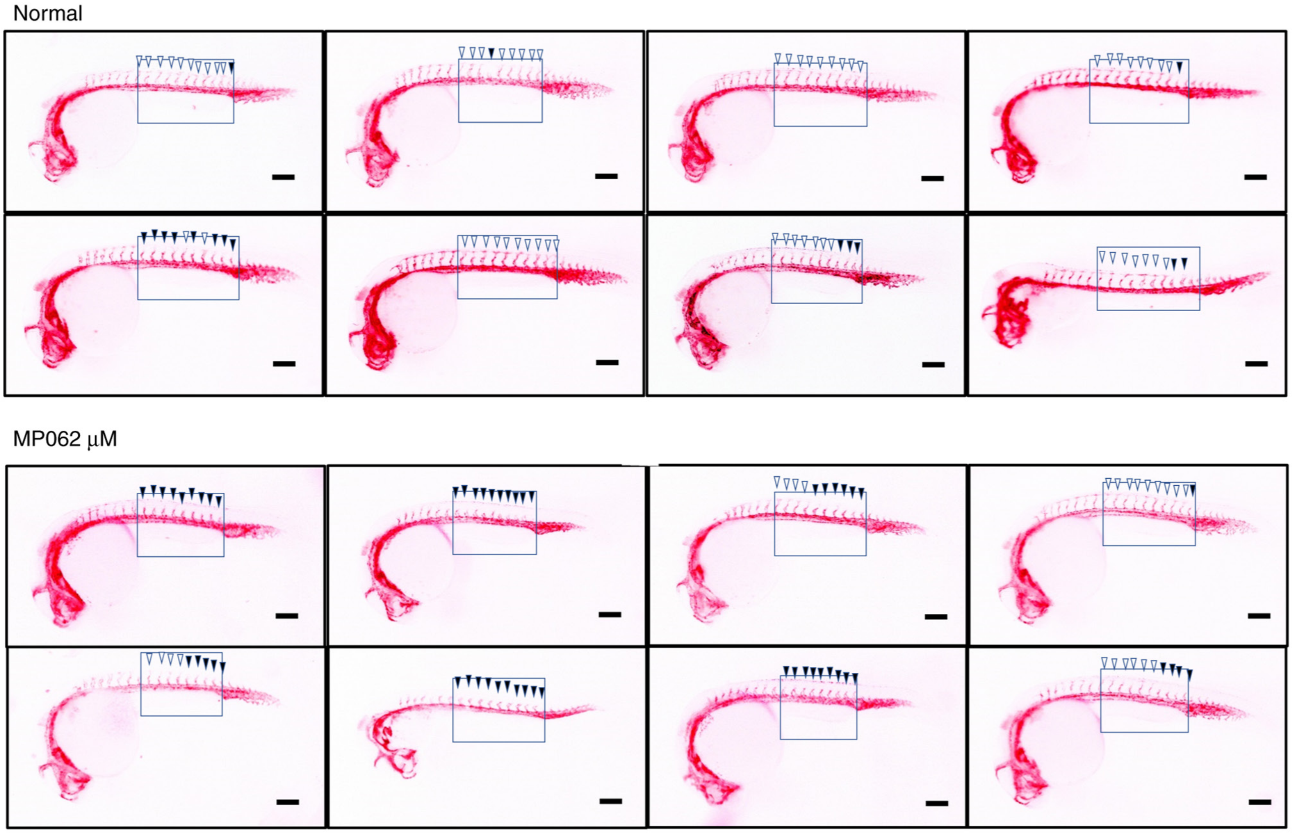

Supplement: Supplementary file 1 [file Supplementary_Data.pdf]
